# Antifungal activity of essential oils against itraconazole-resistant pathogenic *Candida* isolated from vulvovaginal candidiasis

**DOI:** 10.32598/CMM.2023.1359

**Published:** 2022-12

**Authors:** Ali Abdul Hussein S. AL-Janabi

**Affiliations:** 1 Department of Microbiology, College of Medicine, University of Karbala, Iraq

**Keywords:** *Candida*, Essential oil, Itraconazole, Radish, Resistance

## Abstract

**Background and Purpose:**

Fungal infection by species of pathogenic *Candida* with antifungal resistance is currently a serious problem. Treatment with new medications is becoming more challenging to manage this type of infection. The present study aimed to investigate the antifungal effect of essential oils (EOs) against itraconazole-resistant species of pathogenic *Candida*.

**Materials and Methods:**

Seven essential oils were tested on 15 clinical isolates of itraconazole-resistant *Candida* from patients with vulvovaginal candidiasis. The antifungal action of selected EOs was evaluated using the disc diffusion method with the determination of the minimum inhibitory concentration (MIC) of effective Eos.

**Results:**

Radish EO was the most effective type against all *Candida* isolates with MICs between 3.125% and 6.25% (v/v) .It also had a stronger effect than itraconazole. Six other EOs showed antifungal effects at varying concentrations and were dependent upon the type of isolate. Low concentrations of these six EOs were more effective against many isolates than their high concentrations. Moreover, camphor and linseed EOs were less effective on isolates.

**Conclusion:**

Radish EO has a strong antifungal activity against itraconazole-resistance species of *Candida*, even more than itraconazole. The antifungal action of some EOs can be increased through the use of low concentrations.

## Introduction

Many species of *Candida* cause serious infections in immunocompetent and immunosuppressed people with high incidence and prevalence rates [ 1 ].Treatment of these infections with well-known antifungals may promote the development of resistance in different species of *Candida* and non-*albicans Candida* (NAC), which ultimately increases the therapeutic failure of these agents [ 2 ].Therefore, alternative drugs have become an urgent need to control the infection of drug-resistant *Candida* species [ 3 ]. 

Plant components are the best choice at present [ 1 - 2 ]. Essential oils (EOs) are among the main constituents of plants that naturally protect them against biotic and abiotic attacks [ 4 ]. Various EOs have demonstrated antifungal activities against different *Candida spp*. as well as other fungi. Some of these activities may be more significant than those of standard antifungal agents [ 5 ]. Assessment of 38 EOs revealed that 23 of them affected *C. albicans*, while 15 of them were ineffective [ 6 ]. Other studies have also shown that 18 out of 30 types of EOs have antifungal activities against clinical *C. albicans* isolates, compared to12 of them that have no effect [ 7 ]. 

Naturally, about 60 families of plants produce EO [ 8 ]. In this study, the EOs of seven plants (harmal, anise, black seed, cyperus, camphor, linseed, and radish) were selected to evaluate their activity against numerous clinical strains of antifungal-resistant species of *Candida*. The selection was based on the primary indicator of their antifungal activity demonstrated by many previous studies. Results of these studies showed that the components of the selected plants, other than EO, have antifungal effects against *Candida* species. *Peganum harmala* (harmal) seeds, either extracted or not, showed significant inhibitory activity against various species of *Candida* [ 9 - 10 ]. 

Anise liquid extracthas been shown to have antifungal activity against five species of *Candida* and four species of dermatophytes, but no effect on *Candida glabrata* [ 6 ].Moreover, it has been found that the viability of *C. albicans* is significantly affected by increased concentrations of black seed extract [ 11 ].Tuber extracts of Cyperus (Cyperus rotundus), another selected plant, have shown antifungal activity against a number of clinical isolates of *C. albicans* [ 12 ]. 

Regarding the findings of the aforementioned studies, the present research aimed to assess the antifungal activities of the essential oils of seven plants against the pathogenic itraconazole-resistant *Candida spp*.

## Materials and Methods

### Essential oils

Seven types of essential oils (EOs) were selected forthis study. The EOs of harmal seeds (*Peganum harmala* L.; family Zygophyllaceae), anise (*Pimpinella anisum* L.; family Umbelliferae), black seeds (*Nigella sativa* L.; family Ranunculaceae), cyperus (*Cyperus rotundus*; family Cyperaceae), camphor (*Cinnamomum camphora*; family Lauraceae), linseeds (*Linum usitatissimum* L.; family Linaceae), and radish (*Raphanus sativus* L.; Cruciferae) were purchased from Hemeni-Karachi, Pakistan.

### Candida isolates

*Candida* spp. were clinically isolated by vaginal swabs from patients with vulvovaginal candidiasis who had referred to private gynecological clinics in Karbala city, Iraq in February 2021. Specimens were cultured on Sabouraudʼs Dextrose agar (HiMedia, India) and incubated at 35ºC for 24-48 h. Isolated fungi were primarily identified based on morphological characteristics after they were stained with Gram stain. The diagnosis of species was confirmed by the Vitek® 2 system (BioMérieux, France) using Vitek® 2 YST ID diagnostic cards for yeast. Intotal,50 isolates were identified. 

### Antifungal assay

Disc diffusion assay was used to determine the antifungal effect of itraconazole and EOs on the isolated yeast using Clinical and Laboratory Standards Institute(CLSI, M44) methods [ 13 ].A standard itraconazole disc (10 µg, Torrejón de Ardoz, Madrid, Spain) was used to identify antifungal-resistant species of the isolated *Candida*. For EOs, a number of 6 mm diameter discs were prepared from sterilized filter paper. Two concentrations of EO were prepared (100% and 50%, v/v) by dilution with ethanol.

Inoculum of isolated yeasts was prepared to be 1-2×10^8^CFU/ml after adjustment with 0.5 McFarland standard solution. A 100 μl of inoculum was spread by swab on Mueller- Hinton agar (Himedia, India).A disc loaded with EO was added to the inoculated plate and incubated for 24 h at 35°C. Discs with sterilized distilled water and itraconazole were used as controls. The zone of inhibition was determined around the effective disc. The species with a zone of inhibition less than 24-25 mm around the itraconazole disc werereported as resistantspecies [ 14 ].

### Determination of minimum inhibitory concentration of essential oils

The minimum inhibitory concentration (MIC) of the EO in yeast was determined according to the dilution method specified by the CLSI(M27) [ 15 ]. Isolated yeasts were sub-cultured in Mueller-Hinton Broth (MHB) (Himedia, India) for 24 h at 35°C. Serial double dilutions of EO were prepared (3.125%, 6.25%, 12.5%, 25%, 50%, and 100%; v/v).

A plastic microdilution plate (96 wells) was used to determine the MIC value of EO. Afterward, 100 µl of the standard suspension of yeast was added to a well, followed by the addition of 100 µl of each concentration of EO. Several controls were used within a microdilution plate, including MHB alone or with either yeast or EO. The inoculated plate with yeast was incubated at 35°C for 24h. Results were visually read for the presence or absence of growth. 

### Statistical Analysis

Data were analyzed statistically with student t-test using Microsoft Excel for Windows (version 10). It should be mentioned that a p-value of less than 0.05 was considered statistically significant.

## Results and Discussion

In total, 15 out of 50 isolates of *Candida* spp. were found resistant to itraconazole. They included 5,9, and 1 isolates of *C. albicans*, *C. glabrata*, and *C. utilis*, respectively. All of these isolates were used to evaluate their susceptibility to the selected EO. Radish EO was the most effective type of EO for all isolates with a wide zone of inhibition (Table 1). The MIC of radish EO was significantly identified (P=0.03) in most isolates at 6.25%. A strain of *C. albicans*, *C. utilis*, and two of *C. glabrata* revealed susceptibility to low MIC (3.125%) of radish EO (p=0.01) (Table 2). Therefore, it can be said that the effect of radish EO is more significant than that of itraconazole (P=0.01) (Table 1). 

**Table 1:** Susceptibility of isolated *Candida* species to essential oil

Fungal isolate	Zone of inhibition (mm)														
	Essential oil concentration (%)														
	Harmal		Radish		Black seed		Anise		Cyperus		Camphor		Linseed		Itraconazole (10 µg)	
	100	50	100	50	100	50	100	50	100	50	100	50	100	50	
*C. albicans*	15	-	40	40	-	-	-	-	-	4	-	9	-	7	9
*C. albicans*	-	-	35	25	-	2	-	4	-	4	-	-	-	-	10
*C. albicans*	-	-	33	25	-	-	-	-	-	2	-	-	-	4	10
*C. albicans*	-	-	30	25	-	-	-	-	-	-	-	-	-	-	11
*C. albicans*	-	-	25	20	-	-	-	-	-	-	-	-	-	-	12
*C. glabrata*	-	-	40	25	-	4	-	5	-	3	-	2	-	-	8
*C. glabrata*	-	7	30	25	-	12	-	-	-	-	-	-	-	-	7
*C. glabrata*	-	-	30	15	-	-	-	-	-	-	-	-	-	-	7
*C. glabrata*	-	2	40	40	-	-	-	-	-	-	-	-	-	-	7
*C. glabrata*	-	-	25	20	-	-	-	-	-	-	-	-	-	-	8
*C. glabrata*	15	-	40	30	14	-	12	-	10	-	8	-	-	-	9
*C. glabrata*	-	-	30	25	-	2	-	2	-	4	-	-	-	-	7
*C. glabrata*	-	-	40	40	-	4	-	5	-	-	-	-	-	-	10
*C. glabrata*	-	4	30	30	-	6	-	-	-	-	-	-	-	-	10
*C. utilis*	-	8	40	40	-	8	-	4	-	-	-	-	-	-	12

-: there was no effect

**Table 2:** Minimum inhibitory concentration (MIC) of essential oils in an itraconazole-resistant isolate of *Candida* species

Fungal isolate	MIC (%) of essential oil						
	Harmal	Radish	Black seed	Anise	Cyperus	Camphor	Linseed
*C. albicans*	75	3.125	-	-	25	25	25
*C. albicans*	-	6.25	25	25	25	-	-
*C. albicans*	-	6.25	-	-	25	-	25
*C. albicans*	-	6.25	-	-	-	-	-
*C. albicans*	-	6.25	-	-	-	-	-
*C. glabrata*	-	6.25	25	25	25	25	-
*C. glabrata*	25	6.25	6.25	-	-	-	-
*C. glabrata*	-	12.5	-	-	-	-	-
*C. glabrata*	25	3.125	-	-	-	-	-
*C. glabrata*	-	6.25	-	-	-	-	-
*C. glabrata*	75	6.25	75	75	75	75	-
*C. glabrata*	-	6.25	25	25	25	-	-
*C. glabrata*	-	3.125	25	25	-	-	-
*C. glabrata*	25	6.25	25	-	-	-	-
*C. utilis*	25	3.125	25	25	-	-	-

-: there was no effect

The highest level of activity of radish EO for *C. albicans* was also observed in an earlier study at concentrations of 5 and 15 µL [ 16 ]. The biofilm formed on dentures by *C. albicans* was significantly reduced after treatment with radish EO [ 17 ]. However, radish components not only have inhibitory activities against drug-sensitive species, but they can also affect antifungal-resistant fungi, as was observed in the itraconazole- and voriconazole-resistant strains of *Aspergillus fumi gatus*[ 18 ]. 

The chemical analysis of radish EO showed the presence of many chemical components, such as hydrocarbons, fatty acids (i.e., linoleic acid, oleic acid, and stearic acid), aldehydes, esters, ketones, and sulfur-containing substances [ 16 ]. Some of these compounds have antifungal activities, such as caffeic acid and ferulic acid, which have antifungal activities against a number of fungi, including *C. albicans* [ 19 ]. 

In the current study, harmal, black seed, anise, and cyperus EO revealed antifungal properties against many isolates at MIC, ranging from 25% to 75%, while camphor and linseed only affected a few of the isolates. The *C. albicans* was more affected by harmal components, especially harmine, compared to other types of fungi like *Aspergillus niger* [ 9 ].

Clinical isolate of *C. albicans* from the vagina was also inhibited by harmal compounds at MIC 50 ppm and the level of inhibitory action increased with increased harmal concentration [ 10 ]. The EO of harmal consists of more than 105 chemical compounds that are primarily represented by oxygenated monoterpenes, oxygenated sesquiterpenes and eugenol [ 20 ].

Black seed EO has been shown to have an antifungal effect against different types of human pathogenic fungi [ 21 - 22 ]. It has a moderate *in vitro* and *in vivo* effect on different species of *Candida*[ 21 ]. The increase in inhibitory activity against *C. albicans* generally has a direct relationship with the EO concentrations of black seeds [ 22 ]. Therefore, the antifungal action of black seed EO can be useful in reducing the contamination of human food, such as molluscs, with *C. albicans* and other fungi [ 22 ]. The black seed EO consists of at least 14 different compounds, mainly represented by quinones (thymoquinone, thymohydroquinone, and dithy-moquinone), carvone, carvacrol, γ-terpinene, monounsaturated fatty acids, saturated fatty acids (stearic acid and polinic acid), monoterpenes (𝛽-pinene), and sesquiterpenes [ 21 ]. 

Anise EO is another type of EO effective against a wide range of *Candida* isolates from the current study. Many species of pathogenic *Candida* in a previous study were highly inhibited by anise EO at a MIC of less than 1.56% (v/v) [ 23 ]. Anise EO and nine other EOs also showed antifungal activity against many species of *Candida*, depending on the type of EO and fungal species [ 24 ]. Anise EO consists of many chemical compounds represented by 10 main components within the groups of phenylpropanoids and sesquiterpenoid hydrocarbons that all have antifungal action [ 25 ]. 

Many isolates of the present study were more susceptible to low concentrations (50%) of these six Eos, compared to their high concentrations (100%). Their high concentrations were effective on only one or two isolates, while their low concentrations revealed antifungal action against at least two isolates (Table 1).

The compounds of EO occur naturally in a complex mixture with different concentrations [ 4 ]. Dilution of some EOs may enhance the separation of components from their complex mixture [ 26 ]. Therefore, components at high concentrations can remain in the EO after being diluted, even if their concentration has decreased, while low-concentration components can decrease or vanish from the diluted EO. The remaining components in diluted EO may still have antifungal action, even when the EO is used in low concentrations.

Isolate number two of *C. albicans* and two isolates of *C. glabrata* (no. 6 and 11) in the current study were more susceptible to at least four of the EOs. Meanwhile, two isolates of *C. albicans* (no. 4 and 5) and two of *C. glabrata* (no. 8 and 10) were resistant to all tested EOs, with the exception of radish EO (Table 1 and 2).

The antifungal effects of EO against *Candida* spp. may occur through multiple mechanisms of action. Although these mechanisms are not identified for many EOs [ 21 ], They can affect several biological activities in fungal cells, such as inhibition of efflux pumps, biofilm, mycotoxin synthesis, and enzymatic activities for adenosine triphosphatebiosynthesis in mitochondria [ 4 ]. The most important mechanisms of EO action in *Candida* spp. include inhibition of biofilm formation, germination, cell wall integrity, and cell metabolism with increased apoptosis due to loss of cell membrane plasticity [ 5 ]. 

The EO of black seeds has an inhibitory action in many types of yeast by its effects on the plasma membrane, cell wall, nuclei, and mitochondria membranes [ 21 ]. Camphor compound of camphor EO shows fungicidal action by targeting cellular membranes causing the destruction of the plasma membrane and intracellular macromolecules, such as nucleic acids and proteins [ 27 ].

## Conclusion

Essential oils are more efficient to use as an alternative to common antifungals for the treatment of infection with antifungal-resistant species of *Candida*. They contain highly active antifungal compounds and are safe to use. Radish EO has a strong antifungal activity against itraconazole-resistance species of *Candida* even more than the itraconazole effect. The antifungal action of some EOs can be increased through the use of their low concentrations. Species or strains of *Candida* show variable responses to the inhibitory activities of EOs. The application of EOs as a safe therapy for vulvovaginal candidiasis requires further research to be confirmed. Finally, screening for more natural EOs for the treatment of *Candida* infection is advisable.

## Authors’ contribution

A.A.A. contributed to the design, data collection, and writing of the research paper.

## Conflicts of interest

The author states that there is no conflict of interest.

## Ethical Considerations

Ethical approved (IRB) was obtained from the ethical committee of the college of Medicine, University of Karbala with a No. 378 in December 2020.

## Financial disclosure

There is no funding for this research.
